# An Open Multicenter Study of Clinical Efficacy and Safety of Urolastic, an Injectable Implant for the Treatment of Stress Urinary Incontinence: One-Year Observation

**DOI:** 10.1155/2015/851823

**Published:** 2015-04-20

**Authors:** Konrad Futyma, Paweł Miotła, Krzysztof Gałczyński, Włodzimierz Baranowski, Jacek Doniec, Agnieszka Wodzisławska, Maciej Jóźwik, Małgorzata Oniszczuk, Tomasz Rechberger

**Affiliations:** ^1^2nd Department of Gynecology, Medical University of Lublin, Ulica Jaczewskiego 8, 20-954 Lublin, Poland; ^2^Department of Gynecology and Gynecologic Oncology, Military Institute of Medicine, Ulica Szaserów 128, 04-141 Warsaw, Poland; ^3^Department of Gynecology and Gynecologic Oncology, Medical University of Białystok, Ulica M. Skłodowskiej-Curie 24a, 15-276 Białystok, Poland

## Abstract

The prevalence of stress urinary incontinence rises and affects up to 30% of women after 50 years of age. Midurethral slings are currently the mainstay of surgical anti-incontinence therapy. Some patients experience recurrent SUI (RSUI) which is defined as a failure of anti-incontinence surgery after a period of time or persistence of SUI after the procedure aimed at correcting it. The urethral bulking agent application decreases invasiveness of treatment and meets patients requirements. The objective of this study was to assess the safety and clinical efficacy of Urolastic injection. One hundred and five patients with SUI (including 91 patients with RSUI) were treated with Urolastic in three tertiary gynecological clinics. The efficacy of the procedure was assessed objectively at each follow-up visit by means of cough test and a standard 1-hour pad test. Objective success rate after 12 months after primary procedure in RSUI patients was found in 59.3% of patients. In 14 patients with primary SUI improvement after 1 year was found in 71.4% of patients. Although cure rates after MUS are up to 90% there is still place for less invasive treatment option like periurethral injection of bulking agents, especially in patients with previous SUI surgical management.

## 1. Introduction

Stress urinary incontinence (SUI) becomes social disease and affects up to 30% of women after 50 years of age [1, 2]. In addition the prevalence of SUI is increasing, because of rising prevalence of obesity and diabetes mellitus in demographically aging populations of Western world [3]. Although midurethral slings (MUS) are currently the mainstay of surgical anti-incontinence therapy, some patients experience its failures, indicating the need for an appropriate salvage therapy [4, 5]. Moreover, incontinent women expect more and more to be treated with a minimally invasive surgery. The periurethral application of urethral bulking agent (UBA) in local anesthesia decreases invasiveness of treatment and meets patients' requirements [6]. This method should also be developed in order to make treatment possible in people with varied, often live threatening comorbidities, which makes general anesthesia contraindicated. In ageing population it is very important issue to look for future therapies suitable for more demanding patients from medical point of view. The ideal bulking agent should be easily injectable under local anesthesia, non-absorbable, hypoallergenic, nonimmunogenic and it should maintain its shape, volume, and flexibility in order to exert long-lasting clinical effect [6, 7]. Many different bulking materials had been used as bulking agents in the treatment of SUI with long term (2.8 years) improvement rate up to 80% and cure rate up to 40% [8]. Recurrent SUI (RSUI) is defined as a failure of anti-incontinence surgery after a period of time or persistence of SUI after a procedure aimed at correcting it. Moreover, the complication of particular concern after primary or secondary sling is the incidence of voiding dysfunction resulting usually from improper tape positioning or its excessive tension [9, 10]. One has to remember that repeating procedures performed on vaginal skin could cause scarred vagina syndrome. This condition markedly decreases every next vaginal procedure's efficacy in the treatment of stress urinary incontinence and causes periurethral pain syndrome [11]. Urolastic is a new bulking agent used in SUI treatment with success rate up to 68% after one year of follow-up and 30% of minor complications related to the injection [12]. Urolastic is composed of following chemical substances: vinyldimethyl terminated polydimethylsiloxane (PDMS) polymer, tetrapropoxysilane cross-linking agent, platinum divinyltetramethyl siloxane complex as catalyst, and titanium dioxide as a radio-pacifying component. It is used since the 1970s as hysteroscopic tubal plugging in women seeking nonhormonal contraception [13]. Urolastic is injected into the periurethral, submucosal tissue around the bladder neck close to the midurethra. The injection creates increased tissue bulk and subsequent coaptation of the bladder neck and urethra, to achieve a better anatomy, closure of the bladder neck and urethra, thus preventing leakage of urine. The primary objective of the present study was to assess the safety and clinical efficacy of Urolastic injection using Stamey incontinence scale grade [14]. The secondary objective was to evaluate the frequency and severity of any foreseeable complications related to Urolastic.

## 2. Materials and Methods

The study was conducted in accordance with the Declaration of Helsinki, local laws, and regulations relevant to the use of therapeutic agents. Prior to start of the study the protocol was approved by the medical ethics review committee at one of the participating institutions (Warsaw). Between February 2012 and March 2013 one hundred and five patients with SUI (including 91 patients with RSUI) were treated with Urolastic (Urogyn BV, Nijmegen, The Netherlands) in three Polish tertiary referral gynecologic departments. Inclusion criteria for this study were as follows: women with SUI or RSUI as confirmed by medical history and cough test, with at least 2nd grade of incontinence according to Stamey scale, the bladder capacity at least 300 mL or more, and postvoid residual urine of less than 100 mL. Exclusion criteria were detrusor overactivity (DO) or predominately urgency incontinence, pelvic organ prolapse (POP), and suspicion of neurogenic bladder. In RSUI group 77 (85%) of patients had at least one previous midurethral sling surgery, 36 (40%) of them had two previous slings, 9 (10%) had Burch colposuspension, and 5 (5,5%) had anterior colporrhaphy. Mean time from previous surgery in the sling and colposuspension group was 12 months, whereas in colporrhaphy group it was 6 years. Eligible patients were fully informed about the study. The patient received an information sheet and had the opportunity to ask any questions before signing informed consent to participate in the study. Urolastic device consists of a dual container 5 (2 × 2.5) mL syringe. Both ingredients are mixed by means of a static mixer connected to the syringe just before the injection. The bulking material was injected through 18G needle. During injection the syringe is placed in specially designed gun-like injecting device, with ability to inject same amount of Urolastic at each trigger pushing. After injection it becomes permanent and solid. Urolastic was injected, under local anesthesia with 1% lignocaine according to the manufacturer's instructions, at 10, 2, 4, and 8 o'clock positions with 0.5 to 1.25 ccm per spot. If the second injection was needed it was performed 6 weeks after primary procedure and Urolastic was injected only at 4 and 8 o'clock with 0.75 ccm per spot. All injections were performed only by one investigator at each center (KF, JD, and MJ). Immediately after the injection cough test was performed with bladder filled with 200 ccm. Routinely, ciprofloxacin 500 mg bid for 5 days in order to minimize the risk of infection was prescribed. Follow-up visits were scheduled two weeks, six weeks, and 3, 6, and 12 months after primary procedure. The efficacy of the procedure was assessed objectively at each follow-up visit by means of cough test in the supine and standing positions with a comfortably full bladder and a standard 1-hour pad test. A pad weight increase or decrease, when compared to baseline, was then calculated for each patient. Patients were considered completely cured when they were free of all objective SUI symptoms; cough tests as well as a pad test were negative. The procedure was considered as a failure if the patient still reported urine leakage during increases of intra-abdominal pressure, or if the cough tests or pad test was positive. In the improvement group the cough test was negative but patients still reported occasional urinary leakage or the pad test was negative, though the increase in pad weight was minimal: approximately less than 1 gram. Additionally, subjective cure rate was assessed by means of visual analog scale (VAS). Patients had to mark their satisfaction on scaled line with 0–100 endpoints. Stamey incontinence scale was evaluated according to description of the symptoms severity. Statistical analyses were performed with Statistica package version 8.0 (StatSoft Inc., Tulsa, OK, USA). A *P* value < 0.05 was considered statistically significant. Wilcoxon rank test was carried out to test the difference between outcomes of follow-up visits versus baseline characteristics. Intention to treat (ITT) analysis was taken into account when calculating final results of Urolastic efficacy.

## 3. Results

**Figure 1:**
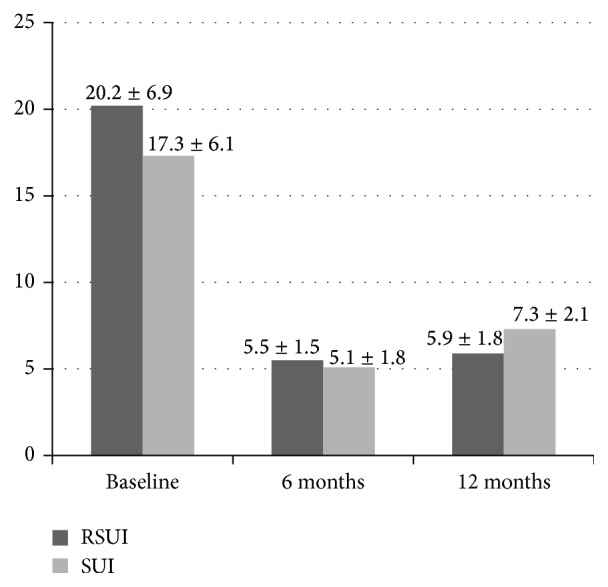
Pad weight test results (g). Change in the pad weight test results: baseline versus 6 months: *P* < 0.01; baseline versus 12 months: *P* < 0.01; and 6 versus 12 months: *P* > 0.07. Wilcoxon rank test, data are presented as a mean ± SD.

**Figure 2:**
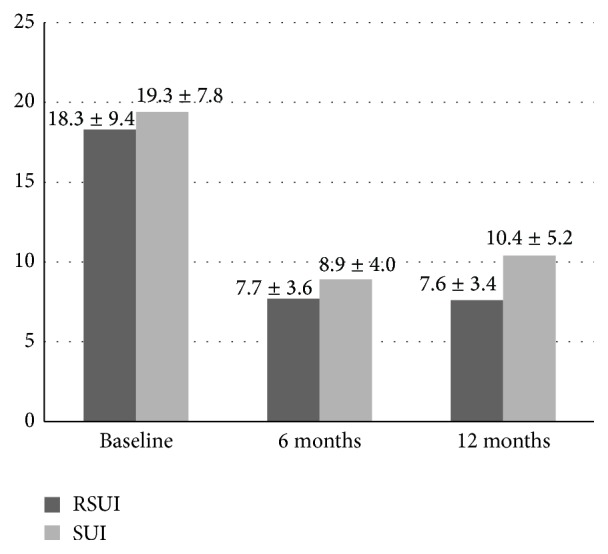
Frequency of incontinence episodes per week (n). Mean numbers of total incontinence episodes: baseline versus 6 months: *P* < 0.01; baseline versus 12 months: *P* < 0.01; and 6 versus 12 months: *P* > 0.05; Wilcoxon rank test, data are presented as a mean ± SD.

**Figure 3:**
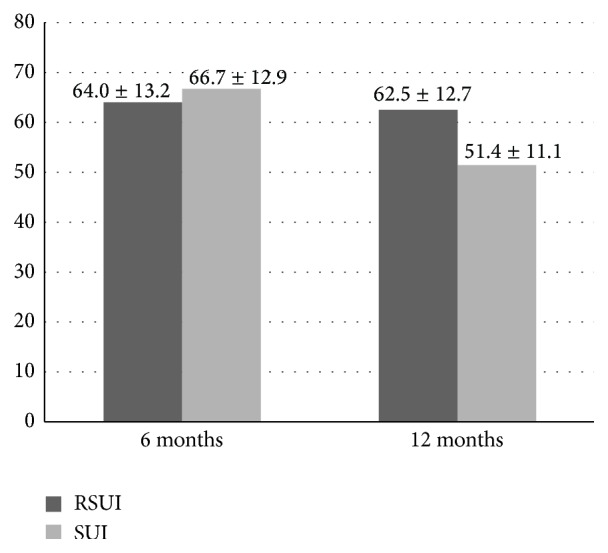
Subjective cure rate as assessed by means of a visual analog scale (VAS), compared to baseline. Subjective cure rate after 6 months: *P* < 0.01 and after 12 months: *P* < 0.001. Wilcoxon rank test, data are presented as a mean ± SD.

Demographic and clinical data of all patients are given in Table 1. Eighty-six patients with RSUI and all treatment-naive patients (*n* = 14) were available for 12-month follow-up, respectively. Eleven RSUI patients and seven treatment-naïve patients required second injection. Objective success rate in patients with RSUI (cured and improved) was found in 54 patients (59.3%) including 45 (49.5%) patients completely dry 12 months after primary procedure. After 1 year, of 14 patients with primary SUI, only 3 patients were totally dry (21.4%), and improvement was found in 10 patients (71.4%). In 10 patients, bladder outlet obstruction (BOO) was observed after injection requiring catheterization for a maximum of 7 days, four of which (40%) required partial removal of the Urolastic material with BOO resolved in all of them. In 4 other patients, some bulking material had to be removed due to its displacement under the urethra which caused pain and dyspareunia. Urolastic was removed during the following surgery (spiral sling). It was very easy to remove as the implants were oval shaped, silicone-like spheres, and we did not observe any incorporation of thematerial into the surrounding tissues. In case when Urolastic was removed from the bladder in other centers we did not hear about any problems with removing the material during cystoscopy from the bladder wall. Three patients experienced recurrent urinary tract infections and were admitted at urology department where some injected material was removed from the bladder during cystoscopy. We did not observe any type of fistula in these patients. No other serious complications including hemorrhage, periurethral abscess, or vaginal wall erosion were observed. Overall, complications in both groups were observed in 17 patients (16.2%). Stamey incontinence grade was significantly decreased compared to baseline, at 6 and 12 months of follow-up after procedure (both *P* < 0.01). Decrease in Stamey incontinence scale by one grade or more was found in 54 (59.3%) RSUI patients and in 10 patients (71.4%) with genuine SUI. Other results after 6 and 12 months are given in Figures 1, 2, and 3.

## 4. Discussion

Published to date clinical results after treatment with UBA are difficult to compare because, first of all, they vary in the bulking agent material, second in patient eligibility criteria, and finally in route of injection [15]. There are few other products on the market today, used to treat female SUI. Most of them are resorbable and thus have ephemeral effect. The first popular product that was used as a UBA was Contigen—collagen material, injected under the urethral or bladder neck mucosa (inside lining) to treat incontinence in men and women. No randomized trials comparing Contigen to conservative therapy or placebo were identified. A randomized clinical trial by Corcos and colleagues compared the efficacy of collagen injections with surgery (Burch colposuspension, needle bladder neck suspensions, and slings) in 133 women [16]. Eligibility criteria included stress incontinence for at least 6 months, or one year after delivery. The twelve-month success rate for collagen treatment was lower than for surgery (53% versus 72%). There were also significantly fewer adverse events in the collagen-treated group (36% versus 63%). Results from this study show superiority of surgery against resorbable bulking agent. In 1999 Durasphere was introduced into the market. A double-blind randomized study comparing carbon-coated beads of zirconium to cross-linked collagen was reported as part of the FDA-approval process. The study showed no difference in efficacy or in the number of treatments between the groups, although the trial length of 12 months may not have been long enough to assess comparative durability [17]. The other study performed to compare the efficacy of calcium hydroxylapatite (Coaptite) with collagen in treatment of SUI showed slight advantage of nonabsorbable material. After the 12 months of follow-up 63% of patients treated with hydroxylapatite and 57% of control patients treated with collagen showed improvement by one grade or more on the four-grade Stamey Urinary Incontinence Scale. Similar results were obtained when ITT analysis was done (58% versus 51%, resp.) and decrease in urine loss by 50% or more in pad weight (51% versus 38%, resp.) was considered [18]. Investigation performed by Ghoniem and coworkers comparing the efficacy of Macroplastique with collagen in women with SUI also showed that nonabsorbable material has higher clinical efficacy compared to absorbable collagen (61.2% versus 48%, resp., *P* < 0.001) [19]. There were no serious treatment-related adverse events reported. The rates of treatment related adverse events are similar between the Macroplastique and the Contigen group, but one exception: the occurrence of postprocedure bladder catheterization is significantly higher among Macroplastique treated subjects (43.4% Macroplastique versus 24.0% Contigen). Two-year data on 67 of 75 women who responded to treatment with Macroplastique were further published in 2010. Fifty-six of the 67 (84%) patients had sustained treatment success at 24 months, defined as an improvement by at least one Stamey Score grade compared to baseline. Forty-five of the 67 (67%) patients evaluated at 24 months were still dry (Stamey grade 0). The interpretation of this long-term outcome is somewhat limited because the analysis included 67 (55%) of 122 patients originally randomized to receiving Macroplastique and did not provide data for the patients in the comparison group [20]. There is limited data about UBA in patients with RSUI. Lee and colleagues published results concerning patients treated with UBA after failed MUS [21]. The cure rate was 34.8% for a median follow-up of 10 months. Surprisingly, 92% of the patients reported a benefit and 77% were satisfied with the treatment. Results of our multicenter study are very promising as they concern the minimally invasive SUI treatment method in patients with a history of failed anti-incontinence surgery history. We need to remember that we had to deal with previously treated patients and each additional procedure in such patients may be not so effective as first one. Although the treatment-naive SUI group was substantially smaller than RSUI group, apparent disproportions in results among the groups can be seen. Improvement was much higher in patients with primary incontinence (71.4% versus 59.3%; *P* = 0.02) but full recovery rate was much higher in the RSUI group compared to treatment-naïve patients (49.5% versus 21.4%; *P* = 0.005). According to the presented data, there is a place for Urolastic—minimally invasive UBA—in the treatment of SUI.

Further research should be conducted to verify the long-term efficacy of this novel and promising bulking agent.

## 5. Conclusions

Although cure rates after MUS are up to 90%, there is still place for less invasive treatment options. Only a carefully selected number of patients will be able to benefit from the periurethral injection of bulking agents, especially patients with previous anti-incontinence surgery. In our opinion the most eligible patients for such therapy are those with low urethral mobility. Higher effectiveness of BA in RSUI patients is probably due to scarred tissue surrounding the urethra which decreases the possibility of injected material displacement over time. The advantage of this method is minimal invasiveness and safety of the procedure.

## Figures and Tables

**Table 1:** Patients' demographic and clinical data.

Parameter	RSUI (*n* = 91)	SUI (*n* = 14)	*P* value
Age at surgery (years ± SD)	63.6 ± 9.4	63.3 ± 14.1	NS
Parity *n* (range)	2.8 (0–6)	2.8 (1–4)	NS
BMI (kg/m^2^ ± SD)	30.1 ± 5.7	30.7 ± 6.7	NS
Stamey Score 2° *n* (%)	45 (49.5)	6 (42.8)	NS
Stamey Score 3° *n* (%)	46 (50.5)	8 (57.2)	NS
Previous anti-incontinence surgeries (mean)	1.41	NA	NA
